# Uterine Balloon Tamponade under Ultrasound Guidance in Women with Postpartum Hemorrhage: A Retrospective Cohort Study

**DOI:** 10.3390/jcm13092632

**Published:** 2024-04-30

**Authors:** Chiara Germano, Flavia Girlando, Andrea Roberto Carosso, Alessandro Messina, Giulia Parpinel, Livio Leo, Rossella Attini, Alberto Revelli, Bianca Masturzo

**Affiliations:** 1 Department of Obstetrics and Gynecology, Nuovo Ospedale degli Infermi, 13875 Biella, Italy; giulia.parpinel@aslbi.piemonte.it (G.P.); rossella.attini@aslbi.piemonte.it (R.A.); bianca.masturzo@aslbi.piemonte.it (B.M.); 2 SCDU2, Department of Surgical Sciences, Sant’Anna Hospital, University of Turin, 10126 Turin, Italy; girlandofl@gmail.com (F.G.); andrea88.carosso@gmail.com (A.R.C.); alessandro.messina@hotmail.it (A.M.); aerre99@yahoo.com (A.R.); 3 Department of Obstetrics and Gynecology, Beauregard Hospital, 11100 Aosta, Italy; lleo@ausl.vda.it

**Keywords:** postpartum hemorrhage, uterine balloon tamponade, ultrasound, uterine atony, Bakri balloon, vaginal delivery

## Abstract

**Background:** Postpartum hemorrhage (PPH) represents one of the principal causes of maternal mortality and morbidity worldwide. Uterine balloon tamponade (UBT) is recommended for the treatment of postpartum hemorrhage due to uterine atony in women who do not respond to pharmacological first-line treatment. The success of UBT in bleeding control is related to the correct positioning of the device, since incorrect insertion can be associated with ineffectiveness and requires time-consuming repositioning maneuvers, with a further increase in blood loss. The use of ultrasound (US) during UBT may increase the success rate by preventing wrong positioning. This study aims to demonstrate the role of US guidance during UBT and to assess whether US guidance can ease positioning and reduce insertion times, preventing additional repositioning maneuvers, in comparison with a US check carried out after balloon insertion. **Methods:** This was a retrospective study including 370 women who underwent vaginal delivery, had PPH caused by uterine atony and required UBT at Sant’Anna Hospital from 2015 to 2019. The exclusion criteria were an abnormal placental site, vaginal or cervical trauma, coagulation defects, uterine anomalies, previous postpartum hemorrhage and previous caesarean section. Included subjects were divided into two groups: the US-guided group (n = 200) underwent Bakri balloon positioning under US guidance, and the non-guided group (n = 170) received a US check only after balloon insertion. The primary outcome was the success rate of the procedure, expressed as the percentage of cases with bleeding control without the need for balloon repositioning; the secondary outcomes were the length of the procedure and some parameters related to blood loss. **Results:** The success rate was 99% for the US-guided group vs. 86% for the non-guided group. None of the patients required hysterectomy. In the US-guided group with respect to the non-guided group, we observed a significant reduction in blood loss (1100 ± 450 vs. 1500 ± 600 mL; *p* < 0.001), Δhemoglobin level (1.8 ± 1.1 vs. 2.7 ± 2.0 g/dL, *p* < 0.001) and time required for the procedure (8 vs. 13 min, *p* < 0.001). **Conclusions:** Our data suggest that the use of US guidance for placement of UBT was associated with reduced need for balloon repositioning, lower blood loss, and faster resolution of postpartum hemorrhage.

## 1. Introduction

Postpartum hemorrhage (PPH), defined as blood loss ≥500 mL after vaginal delivery and ≥1000 mL after caesarean section, is one of the leading causes of maternal mortality and morbidity worldwide [1]. More than 1.5 million women annually face complications related to hemorrhage during pregnancy and postpartum [2], with an increasing trend in high-income countries [3]. Consequently, PPH is receiving increasing attention as a quality indicator for obstetric care. 

Although several risk factors for PPH have been described [4], PPH still occurs even in patients without risk factors [5,6]. The principal causes of PPH are summarized by the “4Ts mnemonic”: tone, trauma, tissue and thrombin [7]. Uterine atony is responsible for at least 70–80% of cases [8]; trauma, including perineal and cervical tears, is a common finding after delivery and, in 20% of cases, leads to massive blood loss; retained placenta or amniotic membranes are involved in 10% of PPH cases, while less than 1% of PPH cases are due to clotting factor deficiencies [9].

Appropriate treatment of PPH includes uterine massage, pharmacological therapy and, in cases of refractory bleeding, uterine balloon tamponade (UBT). Surgical procedures such as hemostatic sutures, vessel ligation and hysterectomy are considered third-level procedures and are indicated in case of failure in bleeding control efforts. UBT is more accessible, less invasive and less expensive than surgical procedures. As no extensive training or complex equipment is required, several international guidelines and obstetric associations support its use [9,10,11,12,13,14,15,16]. Nevertheless, strong evidence regarding the efficacy of UBT is lacking, as randomized studies are scarce and inconsistent, and most data come from observational studies [17]. 

Many types of balloon have been described for the management of uterine bleeding: the Bakri balloon, the condom catheter, the Foley catheter, the Rusch balloon and the Sengstaken–Blakemore tube [18,19]. Some of them (e.g., the Foley catheter, the Rusch balloon and the Sengstaken–Blakemore tube) were proposed with other indications and later used to treat PPH, while others (e.g., the Bakri balloon) were specifically designed to manage PPH. Furthermore, some of these balloons are provided with a dedicated lumen for blood drainage, so that blood loss can be easily evaluated and monitored [20]. In principle, all balloons have the same mechanism of action: after they are positioned at the level of the lower uterine segment and filled with physiological solution, the pression exerted by them overcomes the uterine artery systolic pressure, reducing the blood supply to the myometrium. In view of this mechanism of action, the correct positioning of uterine balloon catheters has pivotal importance: incorrect positioning can be associated with ineffectiveness of the device and requires time-consuming repositioning maneuvers, with a further increase in blood loss.

According to a recent meta-analysis [21], the success rate of UBT is 86% and is comparable to that of surgical treatment, with better performance in the cases of uterine atony, vaginal delivery and placenta previa. The complication rate is rather low (7.6%), including fever or infections (6.5%); endometritis (2.3%); and negative outcomes related to incorrect positioning, such as cervical tears (1.7%), laceration of the lower part of the vagina (4.8%), uterine incision rupture (1.9%) and uterine perforation (2.0%). The infection risk can be reduced by using broad-spectrum antibiotic treatment (e.g., clindamycin plus gentamicin) in case of suspected endometritis or retention of placenta or fetal membranes [9]. 

The risk of incorrect positioning can be reduced by US control: some authors have suggested that US may be used to facilitate the device’s insertion or may be adopted to check its correct positioning at the end of the procedure [22,23]. To date, although ultrasound has been demonstrated to play an important role in the delivery room [24,25,26,27], there is no clear evidence that the positioning of the balloon under US guidance increases the success rate, reducing bleeding, risk of repositioning, or the need for surgical techniques. The American College of Obstetricians and Gynecologists PPH guidelines suggest the use of US guidance during curettage to prevent uterine perforation and to ensure removal of all retained tissue but do not provide indications regarding US control during or after UBT [9]. The aims of this study are to assess the role of US guidance during intrauterine balloon catheter insertion, to determine whether US-guided placement can facilitate positioning, reduce application time and avoid the need for additional repositioning maneuvers.

## 2. Material and Methods

### 2.1. Patients

This retrospective observational cohort study involved women affected by PPH treated with intrauterine double balloon catheter (Bakri post-partum balloon, Cook Women’s Health, Spencer, IN, USA) at the Obstetrics and Gynecology Department of Sant’Anna Hospital from January 2015 to December 2019.

Among 34,417 women who gave birth at Sant’Anna Hospital during the selected timeframe, 623 had PPH after vaginal delivery. Among them, 253 were excluded from the study because their PPH was due to an abnormal placental site, vaginal or cervical trauma, a coagulation defect or uterine anomalies (myomas, uterus didelphys, bicornuate uterus, septate uterus, etc.) or because they had a history of previous caesarean section or previous PPH. Finally, 370 patients with PPH caused by uterine atony were selected. 

### 2.2. Methods

All were treated according to the national guidelines for postpartum hemorrhage [28], including uterotonics as first-line treatment. First, oxytocin was administered as a 5 IU intravenous bolus over 1–2 min; then, maintenance therapy with 10 IU in 250 mL saline was given. During infusion, manual uterine massage was made. Tranexamic acid (1 g in 100 mL saline) was also administered. In case of non-responsive bleeding, a second-line uterotonic treatment including ergometrine (0.4 mg i.m. injection) and/or Sulproston (prostaglandin E; 0.5 mg in 250 mL saline, 0.1–0.4 mg/h) was used. Depending on hemostatic state and hemodynamic changes, fibrinogen or fresh frozen plasma was eventually administered, and blood units were sometimes transfused. The overall blood loss was measured using a graduated plastic collector bag.

Women were considered candidates for UBT when PPH did not respond to first-line management. After insertion, the Bakri balloon was inflated with approximately 300–400 mL of saline, and a piece of gauze was packed into the vagina to prevent displacement. The drainage port of the balloon was connected to a fluid collection bag to monitor bleeding. During balloon catheter insertion, the woman was kept under deep sedation, both because the standard of care for PPH in our department includes curettage of the uterine cavity and with the aim of minimizing discomfort. The balloon was removed, on average, after 12 h. 

As guidelines do not indicate the use of US guidance during balloon catheter insertion as mandatory [28], some operators used US guidance, and some did not; the latter performed US at the end of the insertion procedure to check the correct positioning of the device. This difference in the operators’ choice allowed us to divide patients into two groups according to the timing of US use (during or after insertion). In the US-guided group (n = 200), the insertion of the balloon catheter was performed under US guidance; in the non-guided group (n = 170), the balloon was positioned without US guidance, and the correct placement was checked using US after the procedure and eventually perfected according to the US findings. General Electric’s Logiq p5 Ultrasound Machine (2006, General Electric Company, Boston, MA 02210, USA) was used to perform US.

### 2.3. Statistical Analysis

Clinical data were obtained from a review of the medical records stored in our Archive System (Trakcare^®^, InterSystems Corporation, Cambridge, MA, USA). For each patient, the following variables were registered: age, body mass index, parity, gestational age, duration of Bakri balloon insertion procedure, amount of physiological solution used to fill the balloon, need for Bakri balloon repositioning, total estimated blood loss, Δhemoglobin (difference between circulating hemoglobin levels before and after the procedure), mean hospitalization length, need for admission to the intensive care unit, need for blood transfusion and number of transfused blood units. 

The Bakri balloon success rate was considered the primary outcome; success was defined as the situation in which blood loss stopped with no need for balloon repositioning and in which no accidental displacement of the balloon occurred. The secondary outcomes were all the other abovementioned variables.

According to our national guidelines, studies using anonymized data collected retrospectively are exempted from ethical committee approval. Moreover, all the women who were admitted to our University Hospital signed an informed consent allowing anonymous data collection and analysis.

Statistical analysis was performed using IBM SPSS software (IBM SPSS v.19; IBM Corp., Armonk, NY, USA). Dichotomous data were compared using the χ^2^ test and Fisher’s exact test; continuous data were compared using the *t*-test. ANOVA and the Kruskal–Wallis test were used for discrete variables when appropriate. A *p*-value of <0.05 was considered statistically significant.

## 3. Results

Among 370 patients who faced PPH due to uterine atony and required UBT with a Bakri balloon, 200 (54%) underwent US-guided positioning (US-guided group), while 170 (46%) received US control after the insertion procedure (non-guided group). The demographic characteristics of the patients are reported in Table 1: between the two groups, no significant difference was noted in age, body mass index (BMI), parity or gestational age. 

Outcomes related to the insertion procedure appear in Table 2. The success rate of UBT was overall quite high, and it was never necessary to proceed to hemostatic hysterectomy. However, it was significantly higher for the US-guided group (99.5% vs. 88% in non-guided group), with clear evidence of higher efficacy of the positioning strategy under US guidance. The UBT procedure was significantly shorter in the US-guided group, with the consequence of obtaining significantly lower blood loss and a significantly lower difference between hemoglobin values before and after delivery. Despite this, the proportion of women who required admission to the intensive care unit (ICU) was 3% in both groups, and the need for blood transfusion and the number of transfused blood units were also comparable between the two groups.

In the US-guided group, there was only one case in which the balloon was repositioned after spontaneous dislocation (0.5%), while in the non-guided group, repositioning of the balloon was significantly more frequent (12% of cases). 

## 4. Discussion

### 4.1. Principal Findings

This study demonstrates that US-guided insertion of a Bakri balloon is superior to insertion without US coupled with a post-procedural US check, as it increases the success rate, reduces application time and blood loss and lowers the need for additional repositioning maneuvers. The added value of US guidance is the ability to check the balloon’s position throughout the whole insertion procedure, preventing mispositioning [29] or expulsion during inflation. US guidance makes it possible to see the profile and the angle of the uterus, and, consequently, the insertion of the device into the uterine cavity becomes easier and more accurate, minimizing potential complications. 

### 4.2. Results in the Context of the Literature

According to international guidelines [9,10,13,14,28], UBT has an important role as a first-line surgical treatment after the failure of medical therapy in controlling PPH. Overall, the Bakri balloon is considered quite effective and safe in managing PPH [30,31,32]. However, there are limited data about the optimal technique to insert it and, in particular, about the role of US guidance during insertion [22,23]. 

In all surgical procedures, training is paramount to improve the success of the technique; many authors support the importance of simulation to enhance clinicians’ skills in emergency settings [24,33,34]. Indeed, very realistic mannequins with human features have been created to simulate both clinical maneuvers and US examination during obstetrical emergencies. As the hectic times and the confusion that may occur in the operating room during PPH may hinder the device’s correct positioning [35], training on simulators is very important to acquire the necessary skills. 

According to uterine anatomy, the correct location of the device is at the level of the lower uterine segment, where uterine arteries run to feed the myometrium and decidua [17]. Several balloon-positioning techniques have been described by different authors [12]; the technique adopted herein includes the insertion of the balloon in the uterine cavity, the reduction of the cervical rim with a ring clamp and the subsequent filling of the balloon with 350–450 mL of saline. 

### 4.3. Clinical Implications

The present study suggests that Bakri balloon insertion under US guidance may raise the device’s success rate by providing the correct positioning and is able to reduce the time needed for the procedure, consequently reducing blood loss. Therefore, US guidance significantly facilitates Bakri balloon insertion, even during an emergency: throughout the procedure, the operator can follow the location of the balloon and the filling of the device, feel more confident about the efficacy of the therapeutic strategy and complete the procedure more quickly. In contrast, a US check at the end of the procedure, performed without the help of any specific sonographic landmark, may lead to misinterpretation of the site of the balloon and thus to incorrect positioning, leading, in turn, to repositioning and an increase in blood loss. 

The correct US technique to allow US-guided positioning of the device can be learned during a specific training course, which can even incorporate mannequins designed for this purpose [34]. It is important to locate the probe longitudinally above the pubic bone and then follow the hyperechoic endometrial line without losing sight of it. This technical skill is easy to gain and can even be performed by non-experts [35]. 

Notwithstanding a better safety profile, some studies described the migration or the incorrect positioning of the Bakri balloon even after insertion under US guidance [29]: namely, the device may be involuntary introduced into an unknown myometrial perforation, even if the balloon is placed with US guidance. Obstetricians should be aware of this potential complication because the inflation of the balloon may increase the size of a preexisting uterine hole. A number of potential complications may occur during insertion, including not only perforation but also ulceration of the uterine wall or myometrial rupture due to overdistension [29,35,36]. Women with a previous caesarean section or previous myometrial scars have an increased risk of uterine perforation during Bakri balloon insertion. The US-guided procedure in women with a scarred uterus may be preferred to avoid balloon mispositioning through the previous uterine scar. 

### 4.4. Research Implications

This study represents an early attempt to find a Bakri balloon insertion technique able to enhance its success rate. However, the present findings are only preliminary and need to be confirmed by a prospective randomized controlled trial.

### 4.5. Strengths and Limitations

In this retrospective study, all patients were treated in the same hospital, received the same medical treatment and underwent Bakri balloon positioning with the same insertion technique. This homogeneity of management made it possible to obtain two highly comparable groups. Moreover, the simple size was adequate to observe significant differences in outcome between the groups. The retrospective nature of the study enabled a complete follow-up of each patient. 

By contrast, the retrospective design represents a limitation. Moreover, while the two groups appeared balanced for measured variables, a retrospective study includes the possibility of unmeasured or unknown confounding variables; for example, the choice to apply US during or after insertion was discretionary, depending on the clinician on duty during and after delivery.

## 5. Conclusions

In conclusion, the present study suggests that women with PPH unresponsive to medical treatment may be effectively treated by UBT; a Bakri balloon under US guidance is significantly more effective than insertion followed by US check. Specifically, US guidance is associated with an increase in the success rate of the procedure, a reduction in repositioning maneuvers and a decrease in duration of the procedure as well as blood loss. 

## Figures and Tables

**Table 1:** Demographic characteristics of the two patient groups. n.s.: not significant.

	US-Guided Group n = 200	Non-Guided Group n = 170	*p*-Value
Maternal age (years)	36.7 ± 2.8	35.9 ± 1.6	n.s.
BMI	28 ± 4.8	27.8 ± 4.1	n.s.
Parity nulliparous parous	56 (28%) 144 (72%)	51 (30%) 119 (70%)	n.s.
Gestational age at delivery (weeks)	38.1 (±3.0)	38.6 (±1.9)	n.s.

**Table 2:** Primary and secondary outcomes in the two patient groups. n.s.: not significant.

	US-Guided Group n = 200	Non-Guided Group n = 170	*p*-Value
Mean volume of saline (mL)	325 ± 69	330 ± 52	n.s.
Length of procedure (min)	8 ± 3	13 ± 2	<0.001
Balloon repositioning	1/200 (0.5%)	21/170 (12%)	<0.001
Estimated blood loss (mL)	1100 ± 450	1500 ± 600	<0.001
ΔHb value (g/dL)	1.8 ± 1.1	2.7 ± 2.0	<0.001
Length of hospitalization (days)	5 ± 1	6 ± 2	n.s.
Intensive care unit hospitalization	6 (3%)	5 (3%)	n.s.
Blood transfusion	36 (18%)	42 (25%)	n.s.
Mean units of transfused blood	2	2	n.s.
Success rate	199/200 (99.5%)	149/170 (88%)	<0.001

## Data Availability

The original contributions presented in the study are included in the article; further inquiries can be directed to the corresponding author.

## Abbreviations

PPH: postpartum hemorrhage; UBT: uterine balloon tamponade; US: ultrasound.
